# Asymmetric synthesis of tertiary thiols and thioethers

**DOI:** 10.3762/bjoc.7.68

**Published:** 2011-05-10

**Authors:** Jonathan Clayden, Paul MacLellan

**Affiliations:** 1 School of Chemistry, University of Manchester, Oxford Rd, Manchester M13 9PL, UK

**Keywords:** asymmetric synthesis, organolithium, sulfur, substitution, thiol

## Abstract

Enantiomerically pure tertiary thiols provide a major synthetic challenge, and despite the importance of chiral sulfur-containing compounds in biological and medicinal chemistry, surprisingly few effective methods are suitable for the asymmetric synthesis of tertiary thiols. This review details the most practical of the methods available.

## Introduction

**Figure 1:**
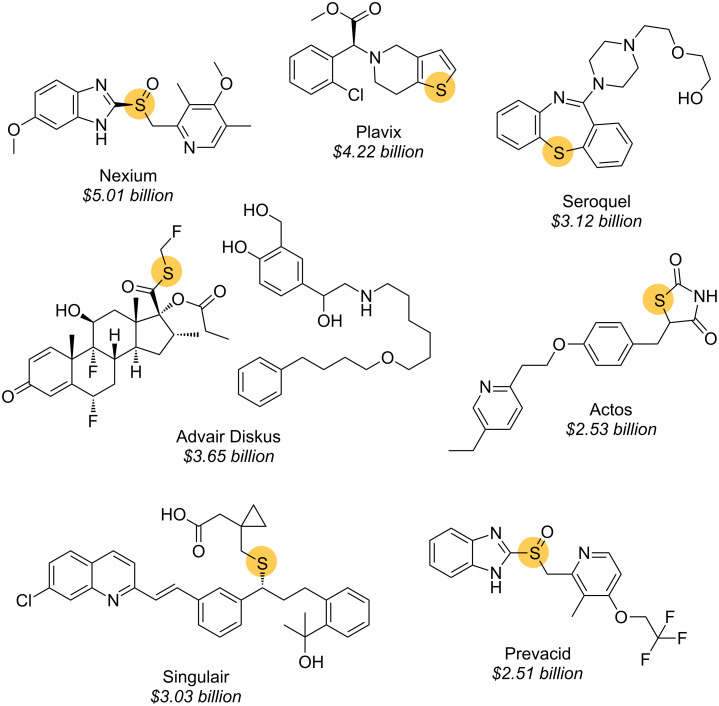
Seven out of the ten top selling drugs in the USA in 2009 contain sulfur. Figures in italics are total retail sales in dollars [1].

**Figure 2:**
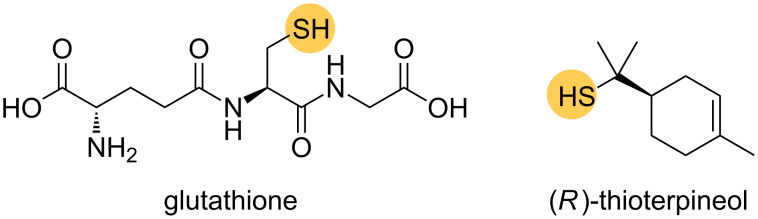
Naturally occurring organosulfur compounds glutathione and (*R*)-thioterpineol.

Organosulfur compounds play key roles in many biological structures and functions: two of the 21 proteinogenic amino acids contain sulfur, and seven of the 10 best-selling drugs in the US in 2009 were organosulfur compounds (Figure 1) [1]. Glutathione plays a crucial role in primary metabolism, and (*R*)-thioterpineol (limonenethiol or “grapefruit mercaptan”) is an important and extremely powerful flavour compound, providing the distinctive taste of grapefruit (Figure 2). The consequent need to prepare and manipulate enantiomerically pure organosulfur species has powered the development of asymmetric synthetic methods leading to various classes of organic sulfur compounds, with chirality residing at sulfur, at carbon, or at both [2–8].

This field of asymmetric organosulfur chemistry is particularly well developed in connection with sulfur(IV) and sulfur(VI) species with chirality at sulfur – namely sulfoxides, sulfinates, sulfimines and sulfilimines [9]. Chiral sulfoxides and sulfonium ylids have themselves been extensively used as tools for asymmetric synthesis [3]. With regard to chiral sulfur(II) compounds – namely thiols and thioethers (sulfides) with chirality at carbon – methods available for their asymmetric preparation are abundant, and usually rely on stereospecific substitution reactions [9]. These reactions are well suited to the construction of *secondary* thiol derivatives. By contrast, few methods are suitable for the asymmetric preparation of simple *tertiary* thiols **1**. While the asymmetric synthesis of simple tertiary *alcohols* is generally achieved by controlling enantiofacial selectivity in nucleophilic attack on a prochiral ketone [10], comparable approaches to tertiary thiols **1** are not practical due to the instability of thioketones, their tendency to undergo nucleophilic attack at sulfur rather than carbon, and their intolerable stench [11]. As a result, enantiomerically pure tertiary thiols are a remarkably difficult class of compounds to make, despite the simplicity of their structure. This review will cover the methods available for the asymmetric synthesis of chiral tertiary thiols and their sulfur(II) derivatives.

**Figure 3:**
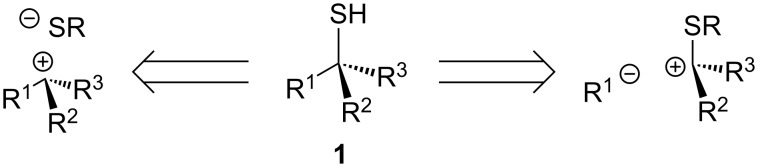
Methods for the synthesis of chiral tertiary thiol **1**.

Two general approaches to the preparation of a chiral tertiary thiol **1** may be envisaged, entailing disconnection of either a C–S or a C–C bond (Figure 3). The thiol could be installed by stereoselective attack of a sulfur-centred nucleophile on a substituted carbon centre (C–S bond formation). Alternatively, stereoselective alkylation, arylation or acylation of a secondary sulfur-based substrate could generate the quaternary centre (C–C bond formation).

## Review

### 1 Carbon–sulfur bond formation

#### 1.1 S_N_2 displacement of a leaving group

Stereospecific nucleophilic attack on substituted carbon atoms is a simple and versatile way to construct stereocentres next to heteroatoms with overall inversion of stereochemistry. Sulfur nucleophiles are commonly used very effectively to accomplish reactions of this type [9]. However, S_N_2 displacements are very sensitive to steric crowding at the reaction centre: S_N_1 substitution and elimination reactions are almost always favoured over the S_N_2 pathway in quaternary electrophiles, limiting the use of S_N_2 reactions for the synthesis of tertiary thiols.

#### 1.1.1 Sulfonate leaving groups

**Scheme 1:**
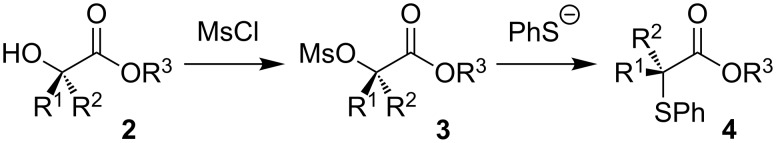
Preparation of thioethers **4** from α-hydroxy esters.

The efficiency of sulfonates as leaving groups has allowed the preparation of certain families of tertiary thiols and thioethers. For example, S_N_2 displacement of a mesylate leaving group by thiophenol can be accomplished using α-hydroxy esters **2** (Scheme 1) [12].

The presence of the α-ester group of **3** promotes S_N_2 reaction in two ways: The electron-withdrawing nature of the substituent inhibits S_N_1 dissociation and carbocation formation, and the planar ester group poses minimal steric hindrance towards approach of the nucleophile.

**Scheme 2:**
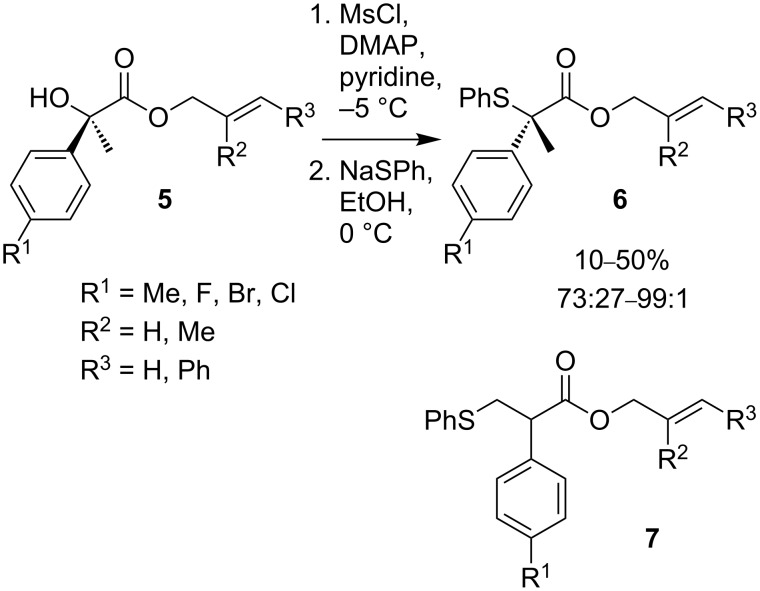
Nucleophilic substitution in α-aryl-α-hydroxy esters.

**Scheme 3:**
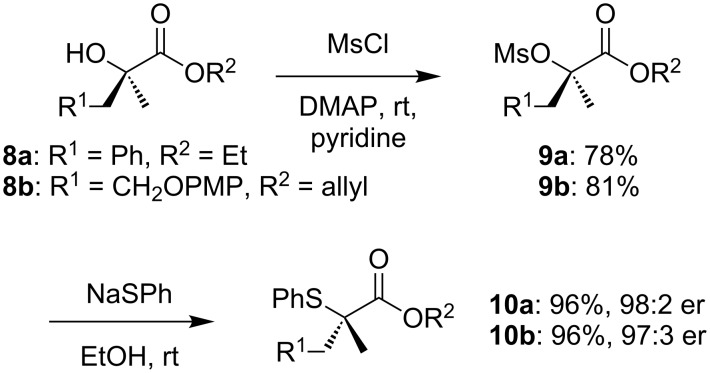
Preparation of α,α-dialkylthioethers.

Yields of substitution in α-aryl-α-hydroxy esters **5** are poor with a significantly decreased enantiomeric ratio in the product **6** (Scheme 2). The low yields can be attributed to the formation of β-phenylthioesters **7** by elimination followed by conjugate addition. Even in the presence of the adjacent carbonyl group, competing S_N_1 dissociation of the benzylic leaving group leads to a loss of enantiomeric purity in some cases. The use of α,α-dialkyl hydroxy esters **8** is more successful: Thioethers **10** are formed in high yield and with almost complete stereospecificity (Scheme 3).

**Scheme 4:**
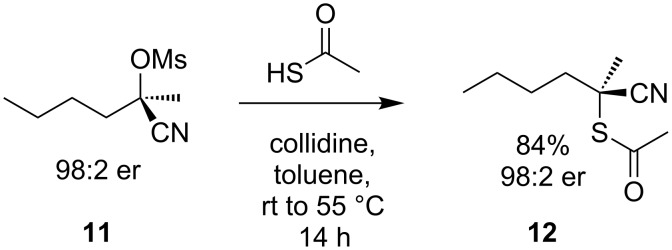
Preparation of α-cyanothioacetate **12**.

Using the same principles of low steric bulk and electronic inhibition of the S_N_1 reaction pathway, α-(sulfonyloxy)nitriles, easily prepared from cyanohydrins, can also be made to undergo S_N_2 reaction with sulfur nucleophiles [13]. However, substitution of the quaternary α-(sulfonyloxy)nitrile **11** is very slow, and conversion of **11** to **12** with potassium thioacetate in DMF proceeds in only 35% yield after 72 hours. S_N_2 displacement of the leaving group by thioacetate in toluene proceeds faster at 55 °C (Scheme 4).

**Scheme 5:**
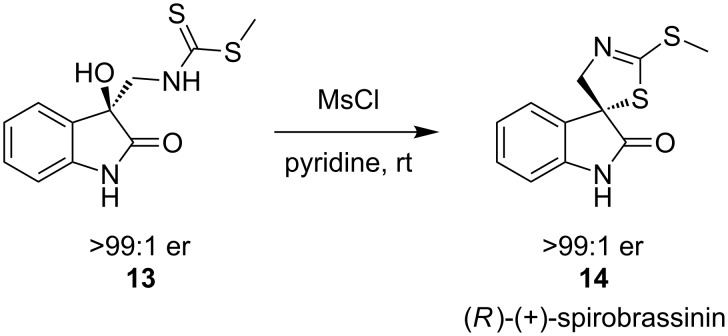
Synthesis of (*R*)-(+)-spirobrassinin.

The synthesis of the unnatural enantiomer of the natural product spirobrassinin has been achieved by substitution at a quaternary centre by a sulfur nucleophile [14]. Intramolecular cyclisation of a dithiocarbamate **13** allows isolation of **14** with complete inversion of stereochemistry, although no yield for this reaction was reported (Scheme 5).

**Scheme 6:**
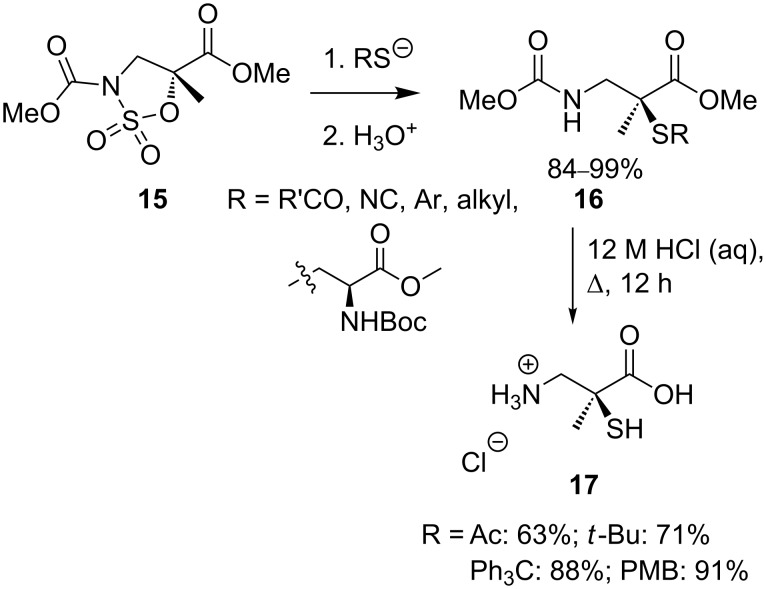
Opening of cyclic sulfamidates with thiol nucleophiles.

Peregrina and co-workers have developed a general method for the synthesis of α-functionalised β-amino acids [15–17] employing nucleophilic ring opening of cyclic sulfamidate **15** via an invertive S_N_2 mechanism (Scheme 6) [16].

A variety of nucleophiles are capable of ring opening the sulfamidate **15** in good yields, and simple functional group transformations allow isolation of the functionalised β-amino acids. Sulfur-based nucleophiles generally undergo reaction with sulfamidate **15** cleanly to give products **16** with complete inversion of configuration [16–17]. Acid catalysed cleavage of a thioether or thioacetate yields *tert-*thiols **17** in good yield.

#### 1.1.2 Epoxide ring opening

**Scheme 7:**
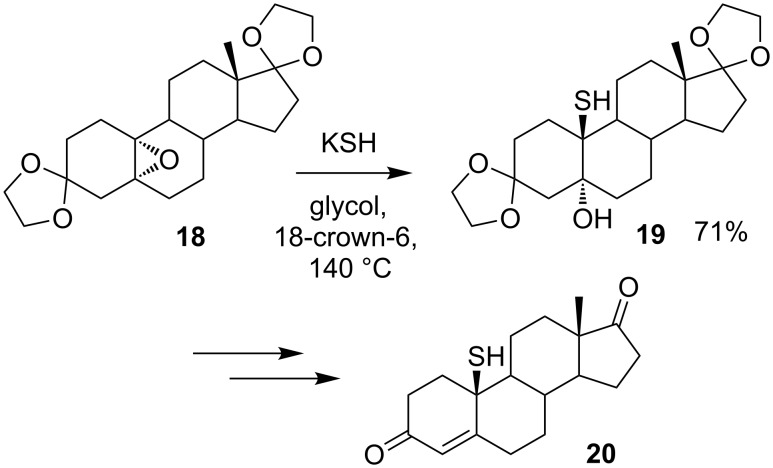
Synthesis of androgen **20**.

S_N_2 displacements from quaternary electrophiles require specific structural features to avoid competing racemisation: Excellent leaving groups and electron withdrawing, non-aromatic and small substituents at the reactive centre all favour invertive substitution. Under basic conditions, epoxides also undergo invertive reactions, even at quaternary centres. Sulfur-based nucleophiles have been employed in nucleophilic ring opening of epoxides in hindered systems [18–23], with most examples of the latter occurring at quaternary carbon atoms which are part of ring systems [18–22], and in particular in steroids [18–20]. For example, the thiol-containing androgen **19** is prepared in 71% yield by epoxide ring opening of **18** with potassium hydrogen sulfide (Scheme 7) [18].

**Scheme 8:**
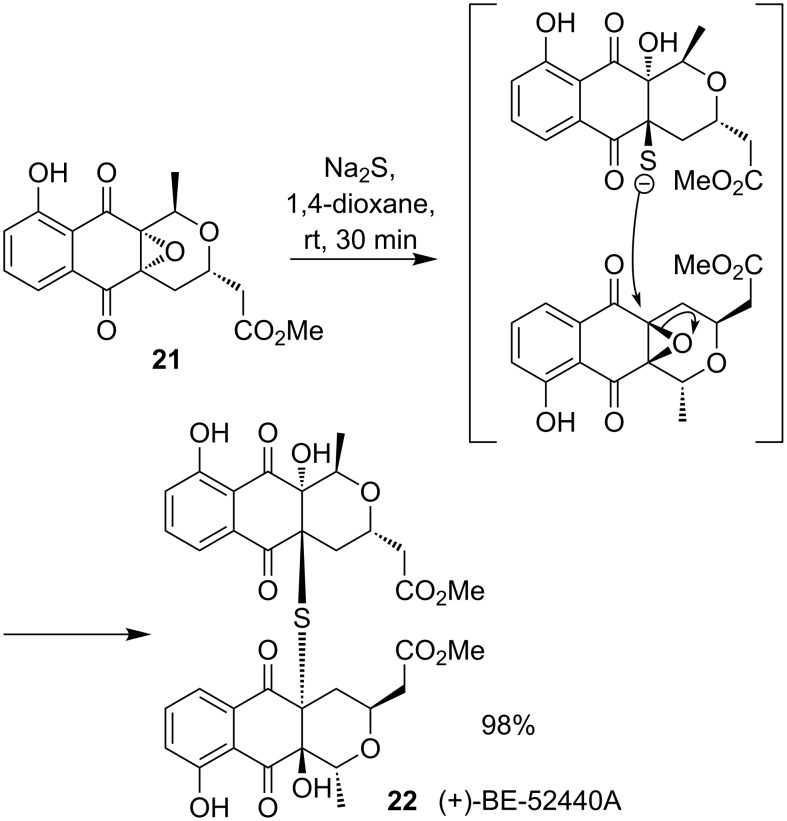
Synthesis of (+)-BE-52440A.

Epoxide ring opening by a sulfur nucleophile was also employed as the key step in the synthesis of (+)-BE-52440A (**22**) [22] (Scheme 8). Dimerisation of nanaomycin derivative **21** through a bridging sulfide involves a double regioselective invertive epoxide opening.

#### 1.1.3 Mitsunobu reactions

**Scheme 9:**
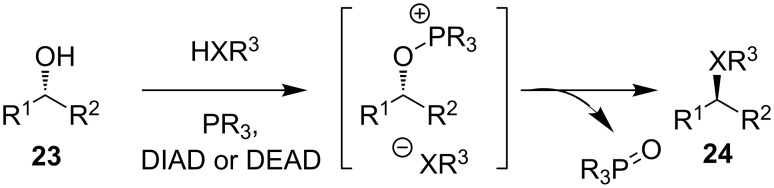
The Mitsunobu reaction.

The Mitsunobu reaction offers an operationally straightforward method for activating simple alcohols to invertive substitution, and it is widely used for constructing new stereodefined carbon–heteroatom bonds [24–25]. The Mitsunobu reaction proceeds by reaction of a phosphine and DIAD or DEAD with an alcohol **23** to form an *O*-phosphinite leaving group (Scheme 9). S_N_2 substitution to yield **24** is accompanied by formation of a phosphine oxide.

**Scheme 10:**
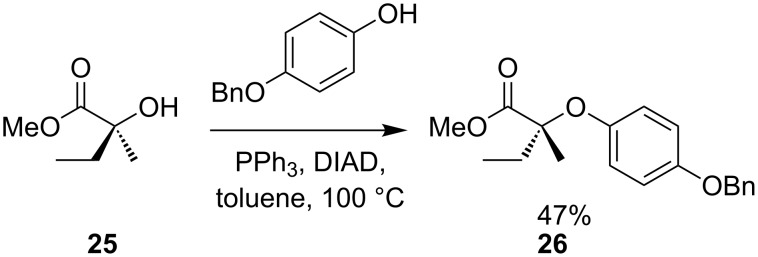
Mitsunobu substitution at a quaternary centre.

There are many examples of the use of sulfur nucleophiles in the Mitsunobu reaction [25–27]: The reaction is successful with a wide range of primary and secondary alcohols, but Mitsunobu-type reactions are very sensitive to steric bulk at the electrophilic carbon atom. For example, reaction of alcohol **25** with a phenol under standard Mitsunobu conditions at 50 °C for 16 hours provides only a trace amount of the desired chiral ether **26**, while reaction at 100 °C allows isolation of **26** in moderate yield (Scheme 10) [28]. A *gem*-diethyl analogue of **25** failed to give any substitution at all, the major product being the result of elimination.

**Figure 4:**
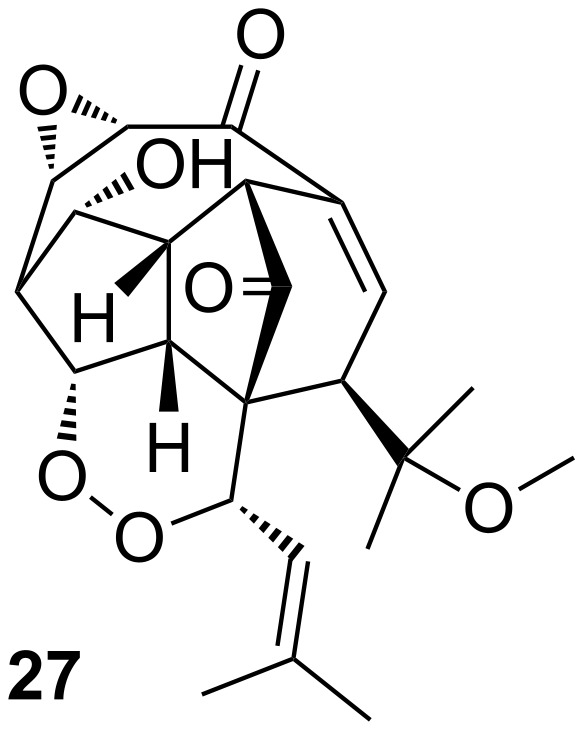
Initially assigned structure of hexacyclinol.

Despite the proven reluctance of tertiary alcohols to undergo Mitsunobu reactions, there is one example of the displacement of a tertiary alcohol by a thiol under Mitsunobu conditions in the literature. La Clair reported the synthesis of the initially assigned structure of natural product hexacyclinol (**27**, Figure 4) [29].

**Scheme 11:**
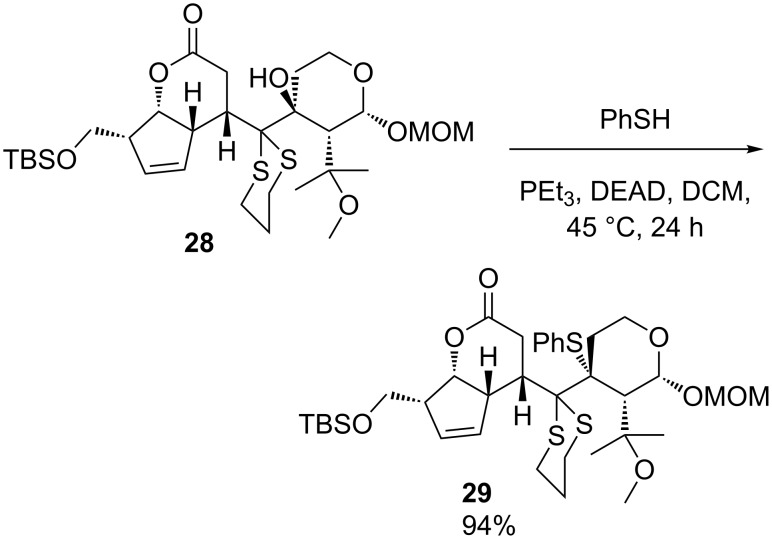
Preparation of thioether **29**.

The reported synthesis entailed Mitsunobu reaction at a very hindered quaternary centre, accomplishing invertive substitution of **28** with thiophenol under mild reaction conditions to yield **29** in 94% yield (Scheme 11).

The paucity of literature precedents for Mitsunobu reactions of tertiary alcohols brings into sharp focus the remarkable success of this reaction under such mild conditions. However, this reaction and other unusual steps in the synthesis attracted scepticism among synthetic chemists [30], and many aspects of this synthesis were called into question when a revised structure of hexacyclinol was proposed by Rychnovsky [31]. A successful synthesis of the revised structure matched the published data for the natural compound [32].

**Scheme 12:**
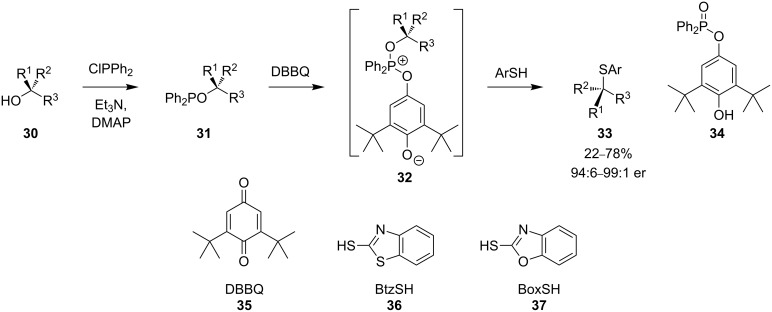
Thioethers **33** prepared from phosphinites **31**.

A modification of the Mitsunobu reaction, that does allow the formation of sulfur-substituted tertiary carbon atoms with inversion of stereochemistry, was reported by Mukaiyama and co-workers [33–34]. The method employs benzoquinone derivatives instead of azodicarboxylates as the oxidising agent. A series of phosphinites **31** was prepared by treatment of alcohols **30** with chlorodiphenylphosphine in the presence of triethylamine and DMAP. S_N_2 substitution of the phosphinites proceeds by oxidative activation of the leaving group with a 1,4-benzoquinone derivative, DBBQ (**35**) being most effective (Scheme 12). Addition of a thiol nucleophile to adduct **32** results in S_N_2 inversion and isolation of the enantiomerically pure (94:6–99:1 er) tertiary thioether **33** and by-product **34**. Highest yields were obtained with BtzSH (**36**) and BoxSH (**37**).

**Scheme 13:**
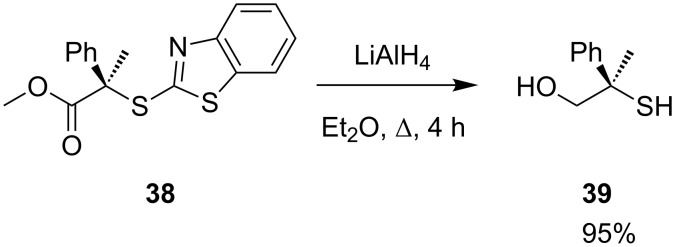
Preparation of enantiomerically pure thiol **39**.

The reaction proceeds well with many hindered substrates incorporating aromatic, alkyl and ester substituents with excellent stereospecificity. Enantiomerically pure thiols can also be made from the product: Aromatic thioether **38** is reduced with lithium aluminium hydride to give **39** in 95% yield (Scheme 13).

**Scheme 14:**
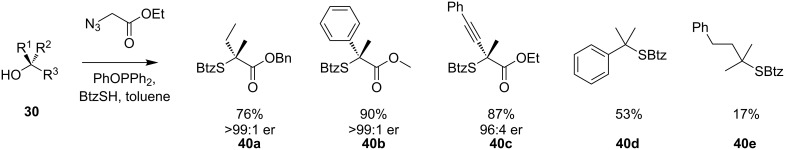
Thioethers prepared by a modified Mitsunobu reaction.

The methodology was later refined to allow substitution of alcohols in 1 step. Phenoxydiphenylphosphine was used to prepare the phosphinite intermediate and an azide was used as the oxidising agent instead of a benzoquinone derivative (Scheme 14) [35–37].

These conditions allow the efficient preparation of chiral tertiary thioether products **40** from hindered tertiary alcohols. However, the yield of the simple alkyl-substituted thioether **40e** was a disappointing 17%. As with other methods, the preparation of simple optically pure chiral tertiary thiols by Mitsunobu-type procedures is not straightforward. Mitsunobu reactions also suffer from atom inefficiency due to the stoichiometric quantities of phosphorus-containing by-products produced by the reaction.

#### 1.2 Conjugate addition

**Scheme 15:**
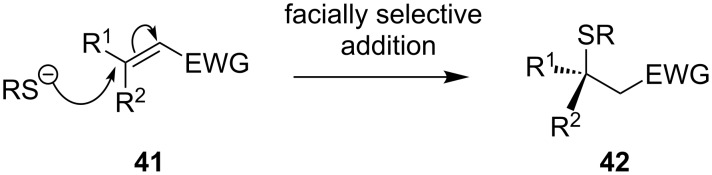
Nucleophilic conjugate addition.

The carbon–sulfur bond of enantiomerically pure tertiary organosulfur compounds **42** may be constructed by facially selective addition of a sulfur nucleophile to the disubstituted terminus of a conjugated alkene **41** (Scheme 15).

There are several examples of such facially selective addition of thiols to substituted sp^2^ carbon atoms, including intramolecular conjugate addition in steroidal systems [38–39] and intermolecular addition directed by substrate stereochemistry [40–41] or a chiral auxiliary [42–43]. Despite these examples, there are very few general procedures for the preparation of chiral tertiary thiols and thioethers by this method. This may be attributed to three difficulties: The low reactivity of β,β-disubstituted Michael receptors, the difficulty in controlling π-facial stereoselectivity, and the equilibrium of stereoisomers through an addition/elimination mechanism.

**Scheme 16:**
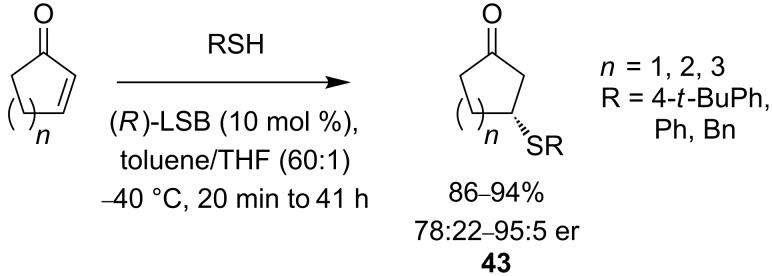
Asymmetric addition to cyclic enones.

The reduced reactivity of β,β-disubstituted Michael receptors towards nucleophilic addition is demonstrated by the catalytic asymmetric additions of thiols to α,β-unsaturated ketones [44]. (*R*)-LaNa_3_tris(binaphthoxide) (LSB) catalyses asymmetric addition to cyclic enones (Scheme 16), and products of addition **43** are isolated in good yields and enantiomeric ratios.

**Scheme 17:**
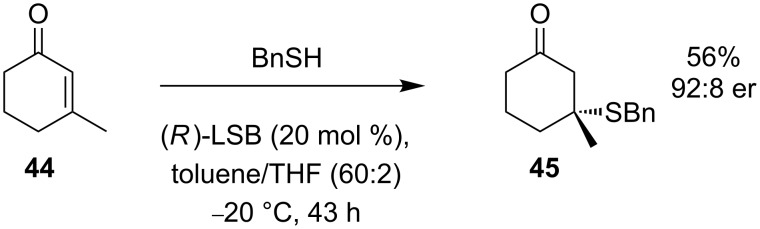
Preparation of thioether **45**.

However, addition to the substituted substrate **44** is more difficult. A higher loading of the catalyst and higher temperature are required to produce the enantioenriched thioether **45** even in moderate yield and acceptable er (Scheme 17).

**Scheme 18:**
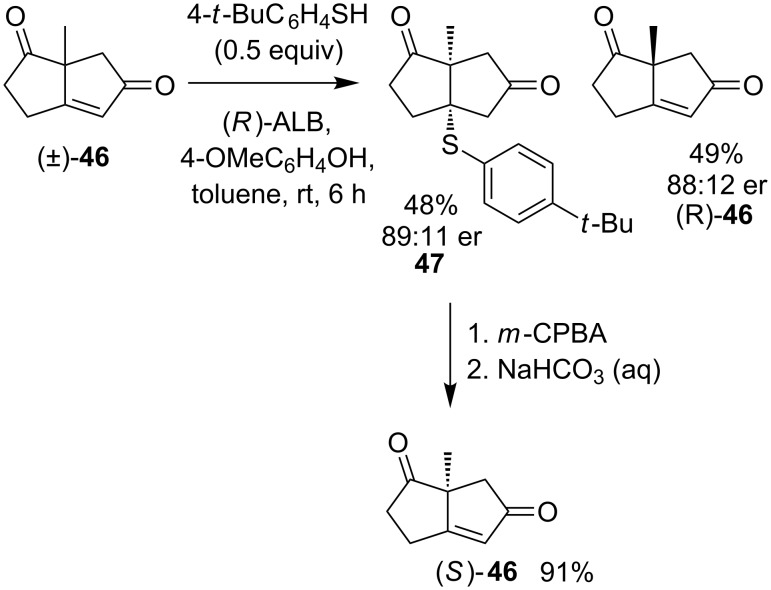
Catalytic kinetic resolution of the enantiomers of enone **46**.

Despite the poor yield obtained with this substrate, similar conditions lead to catalytic kinetic resolution of the enantiomers of hindered enone **46** by addition, oxidation and elimination of a sulfenic acid under basic conditions (Scheme 18) [45].

**Scheme 19:**
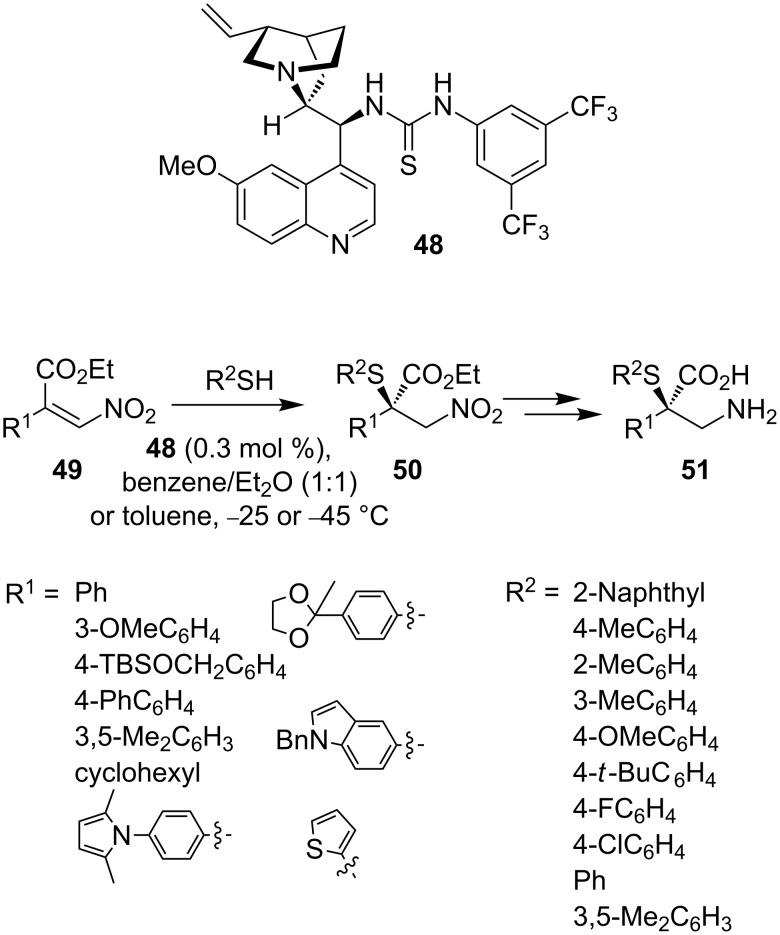
Organocatalytic conjugate addition to nitroalkenes **49**.

Xiao and co-workers developed an organocatalytic process for the addition of thiols to nitroalkenes [46]. Using thiourea organocatalyst **48**, conjugate addition of a variety of thiols to a range of nitroalkenes **49** proceeds to give **50** and hence **51** in good yield and good enantioselectivity (Scheme 19).

**Scheme 20:**
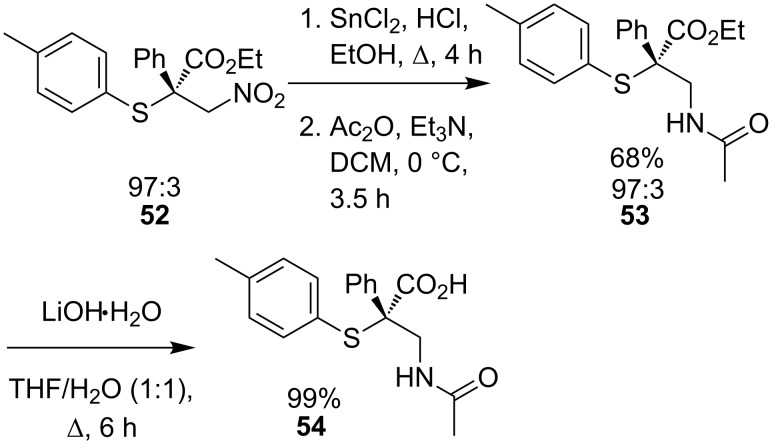
Preparation of β-amino acid **54**.

α-Arylthio-β-amino acid **54** was prepared from Michael adduct **52** in three steps (Scheme 20) in good yield and with full conservation of enantiomeric purity.

**Scheme 21:**
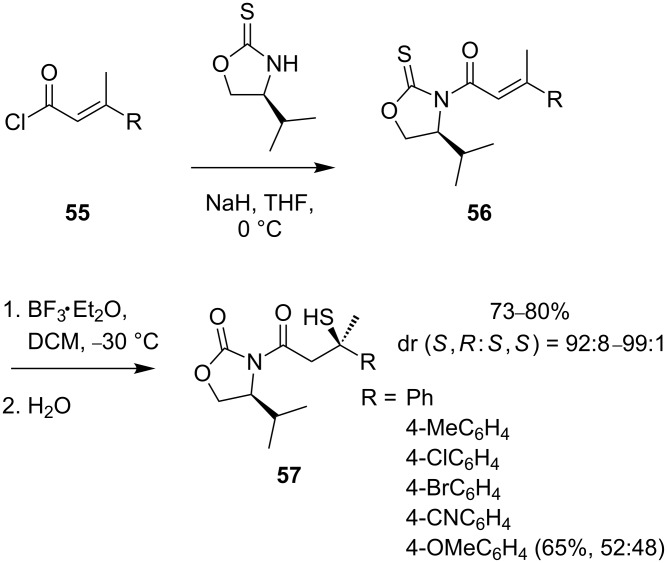
Sulfur migration within oxazolidine-2-thiones **56**.

Palomo and co-workers postulated that poor reactivity and facial selectivity in conjugate additions with sulfur could be minimised by an intramolecular approach [47–48]. A Lewis acid-promoted sulfur migration within *N*-enoyl oxazolidine-2-thione substrates **56** followed by hydrolysis gave optically pure tertiary thiols **57** directly (Scheme 21), with only the electron rich *para*-methoxyphenyl substituent performing poorly.

As with stereospecific nucleophilic displacement, only specific substrates are tolerated in conjugate additions, and further functionality is always required to activate the electrophilic alkene towards attack by the sulfur nucleophile.

### 2 Carbon–carbon bond formation

#### 2.1 Electrophilic addition to α-thioenolates

**Scheme 22:**
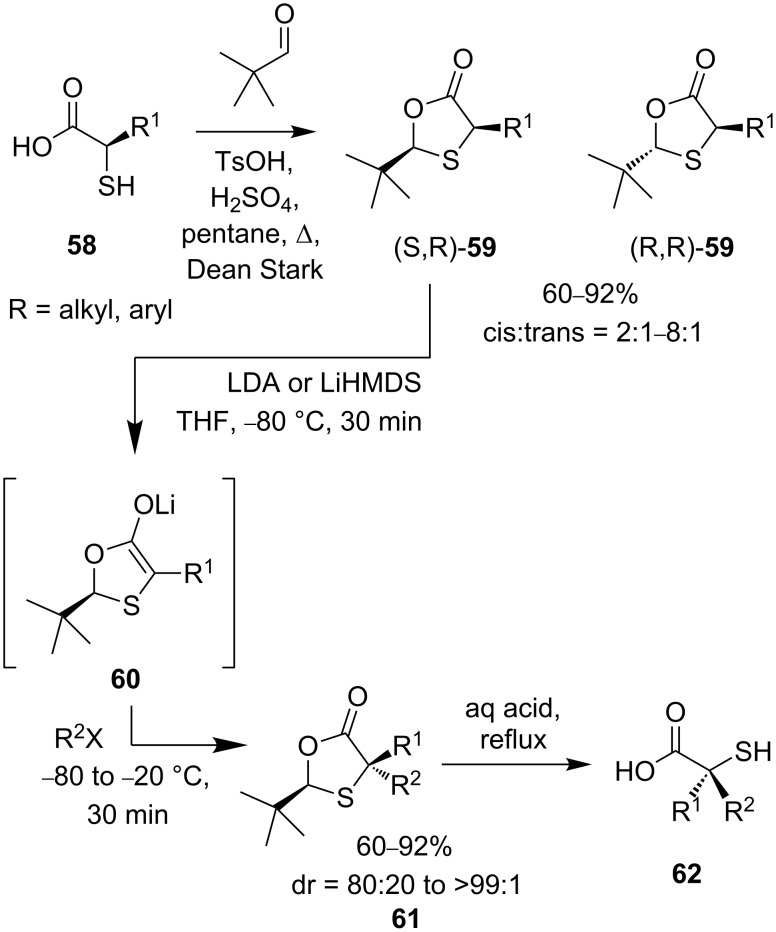
Preparation of thiols **62** by self-regeneration of stereocentres.

Diastereoselective alkylation of α-thioenolates has been used to prepare several types of enantiomerically pure tertiary thioethers [49–54]. The method of “self-regeneration of stereocentres” developed by Seebach [55] employs α-thiocarboxylic acids **58** condensed with pivaldehyde to generate 1,3-oxathiolan-4-ones **59** (Scheme 22) [49–50]. The *cis* diastereomer is formed preferentially (2:1–8:1 selectivity), and can usually be purified by crystallisation. Enolate **60** is formed by deprotonation with a lithium base and treated with an electrophile. Electrophilic addition takes place diastereoselectively anti to the bulky *tert*-butyl group, producing oxathiolanone products **61** in excellent diastereoselectivities.

**Scheme 23:**
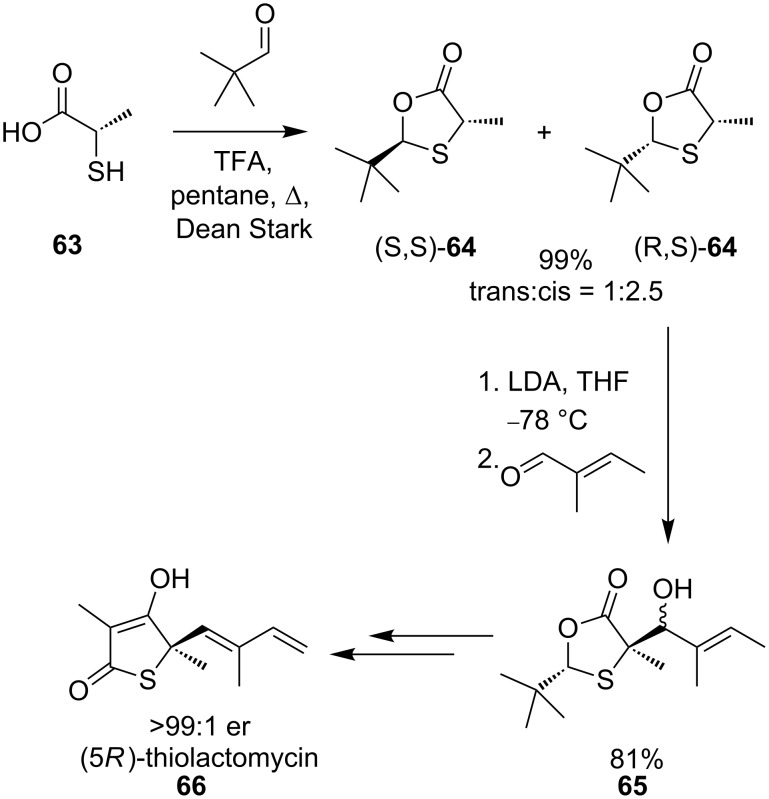
Synthesis of (5*R*)-thiolactomycin.

Enantiopure tertiary thiols **62** can be liberated from the oxathiolanone products by hydrolysis, and the method was employed by Townsend and co-workers in the synthesis of (5*R*)-thiolactomycin (**66**, Scheme 23) [51]. Diastereoselective addition of an aldehyde to oxathiolanone **64** affords **65** in 81% yield. Further synthetic manipulations allowed preparation of (5*R*)-thiolactomycin (**66**) in >99:1 er.

#### 2.2 Electrophilic attack on α-thioorganolithiums

**Scheme 24:**
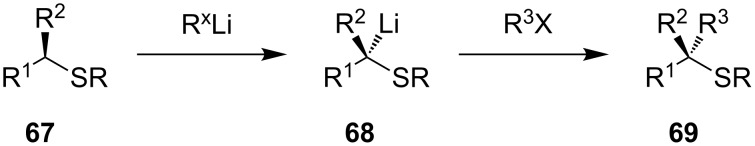
Preparation of tertiary thiols and thioethers via α-thioorganolithiums.

Formation of carbon–carbon bonds adjacent to heteroatoms by deprotonation with an organolithium base and subsequent reaction with an electrophile has become an important and versatile method, especially when chiral ligands may be used to govern the enantioselectivity of the reaction pathway [56]. Tertiary organosulfur compounds can be made in this way by lithiation of a chiral secondary thiol derivative (Scheme 24).

Three conditions must be met if this method is to be used for preparation of enantiomerically pure tertiary thiol derivatives:

1) The substrate **67** must be available in an enantiomerically pure form,

2) The lithiated intermediate **68** must be configurationally stable, and

3) Electrophilic addition to give **69** must proceed with either complete retention or complete inversion of stereochemistry.

#### 2.2.1 Configurational stability in α-thioorganolithiums

**Scheme 25:**
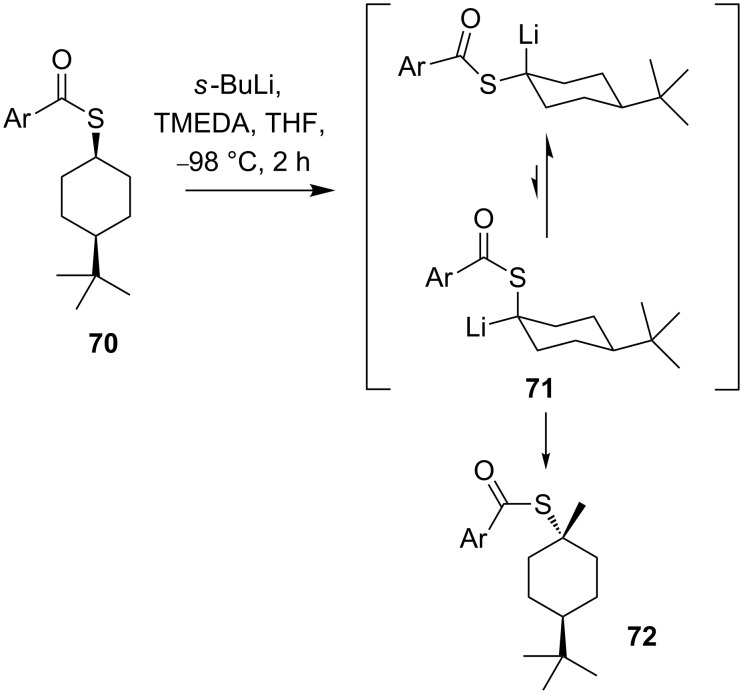
Diastereoselective methylation of organolithium **71**.

In contrast to α-oxy and α-aminoorganolithiums, simple α-thioorganolithium compounds show high configurational lability even at –80 °C [57]. Beak noted configurational instability during the diastereoselective methylation of α-thioorganolithium **71** (Scheme 25) [58]. Equilibration of the diastereoisomers of **71** favoured axial methylation of the lithiated intermediate to give **72**.

Studies by both Hoffmann and co-workers [59–60] and by Reich and co-workers [61] showed that the rate determining step for racemisation of α-thio, α-seleno and α-telluroorganolithiums is rotation about the C–S, Se or Te bond [62]. Simple inversion in α-thio-substituted carbanions has a barrier as low as 0.5 kcal·mol^−1^ [63] making inversion itself unlikely to comprise the rate determining step of the racemisation. Consistent with this explanation, the barrier to racemisation increases with greater steric bulk in the chalcogen substituent.

#### 2.2.2 Lithiation and alkylation of thiocarbamates

**Scheme 26:**
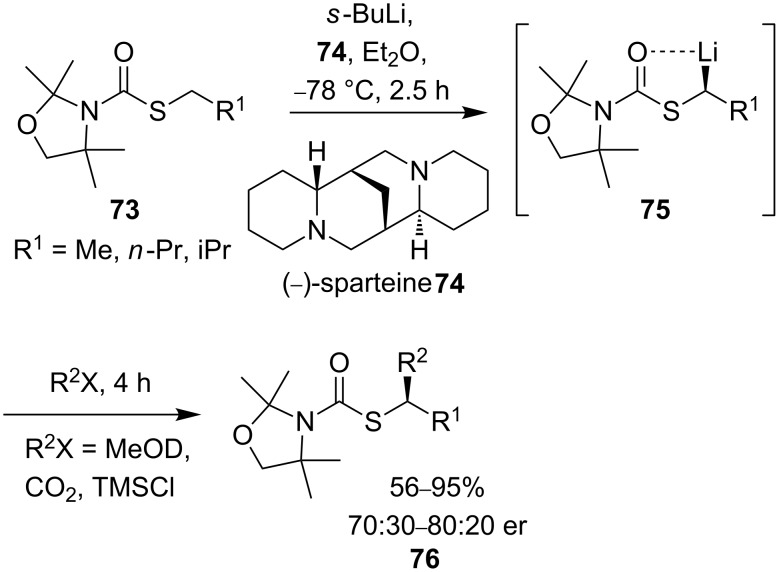
Addition to lithiated thiocarbamate **75**.

Hoppe and co-workers applied the observations of Hoffmann and Reich in comprehensive studies on configurationally stable α-lithiothiocarbamates. Stereoselective deprotonations of thiocarbamate **73** in the presence of (−)-sparteine **74** (Scheme 26) were attempted [64].

**Scheme 27:**
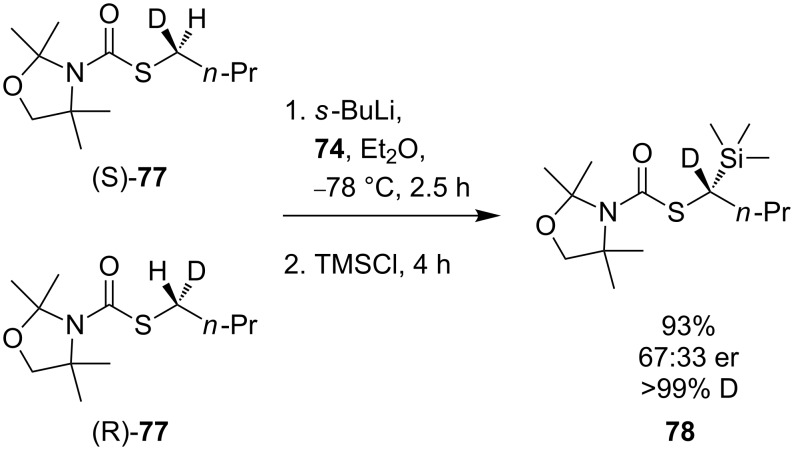
Configurational lability in unhindered α-lithiothiocarbamates.

Trapping the lithiated intermediate **75** with electrophiles gave products with poor enantioselectivities, but the results gave no indication of the configurational stability of the intermediate organolithium **75**. To establish the factors governing enantioselectivity in the electrophilic substitution of **73**, Hoppe made use of an extremely high kinetic H/D isotope effect observed for deprotonation in similar compounds [65]. Reaction of a test substrate demonstrated an isotope effect of *k*_H_/*k*_D_ ≥ 100. A racemic mixture of **77** was lithiated in the presence of (−)-sparteine and quenched with trimethylsilyl chloride (Scheme 27). Deuterated thiocarbamate **78** containing >99% deuterium was isolated from the reaction with an enantiomeric ratio of 67:33. The high deuterium content of the product indicated that both enantiomers of **77** must have been deprotonated, and thus that the lithio derivative of thiocarbamate **77** must be configurationally labile since the product is non-racemic.

**Scheme 28:**
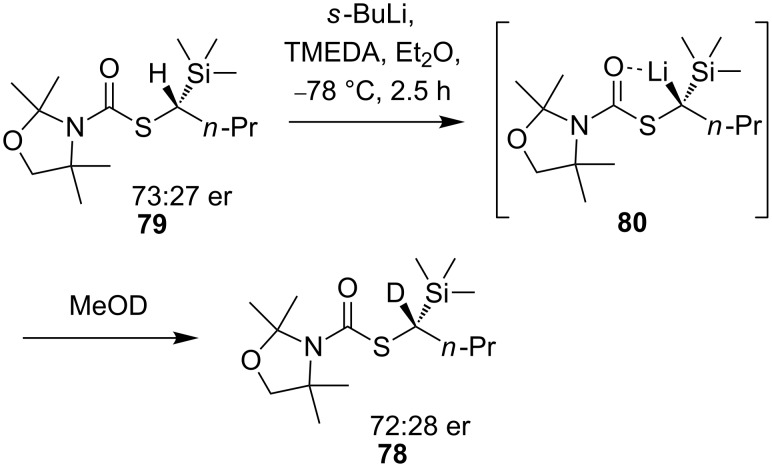
Configurational stability in bulky α-lithiothiocarbamates.

In order to induce greater configurational stability, thiocarbamate **79**, with increased steric bulk, was prepared. A sample of **79** with an enantiomeric ratio of 73:27 was lithiated with *sec*-butyllithium in diethyl ether and TMEDA at −78 °C for 2.5 hours and quenched with MeOD (Scheme 28). Deuterated thiocarbamate **78** was isolated with an enantiomeric ratio of 72:28, indicating almost full conservation of enantiomeric purity and hence demonstrating that the more congested organolithium **80** is now configurationally *stable* under the reaction conditions. Enantiomerically pure tertiary thiols can be liberated from the substitution products by cleavage of the carbamoyl functionality, providing a rare asymmetric route to simple unfunctionalised tertiary thiols.

**Scheme 29:**
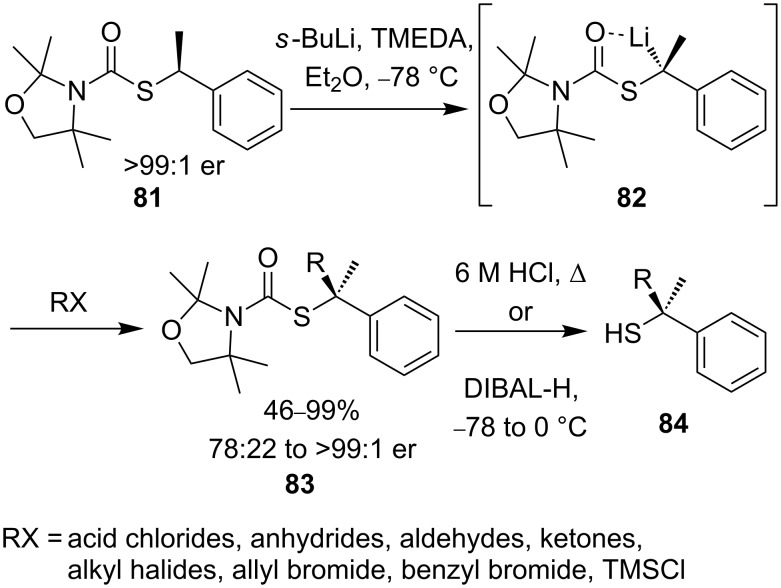
Asymmetric functionalisation of secondary benzylic thiocarbamates.

The lithio derivative of benzylic thiocarbamate **81** was also configurationally stable in diethyl ether and TMEDA at −78 °C [66–67]. Reaction with a series of electrophiles allowed isolation of functionalised thiocarbamates **83** in excellent yield and enantiomeric ratio (Scheme 29). Benzylic organolithiums are notorious for the lack of consistency with which they undergo electrophilic substitution [56], and **82** is remarkable in that all electrophiles, except protonating agents, react with complete *inversion* of configuration.

**Scheme 30:**
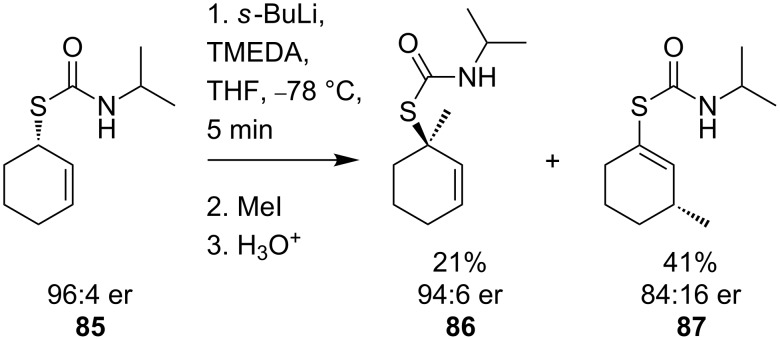
Methylation of lithioallyl thiocarbamates.

Enantiopure cyclohexenyl thiocarbamates also form configurationally stable α-thioallyllithium species [68–70]. Lithiation of thiocarbamate **85** followed by methylation results in the isolation of regioisomeric products arising from electrophilic substitution at either end of the lithioallyl system (Scheme 30).

**Scheme 31:**
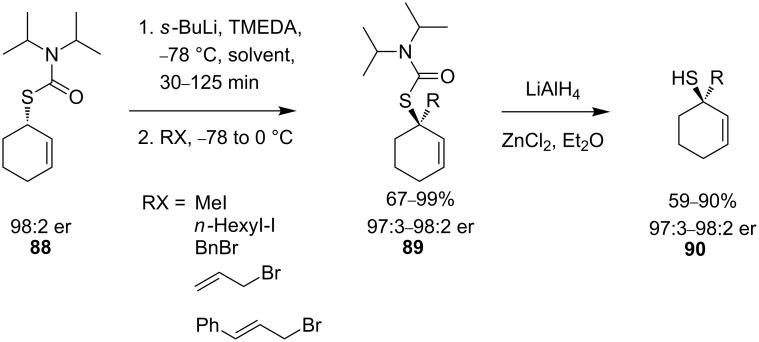
Asymmetric preparation of tertiary allylic thiols.

In the case of the secondary thiocarbamate, the γ-substituted product **87** is preferentially formed, whilst with an *N*,*N*-diisopropylthiocarbamate selective formation of the α-substituted product **89** (Scheme 31) is observed.

Reduction of the functionalised thiocarbamate products **89** with lithium aluminium hydride affords enantiomerically pure thiols **90** in excellent yields and enantiomeric ratios.

#### 2.2.3 Lithiation and rearrangement of thiocarbamates

**Scheme 32:**
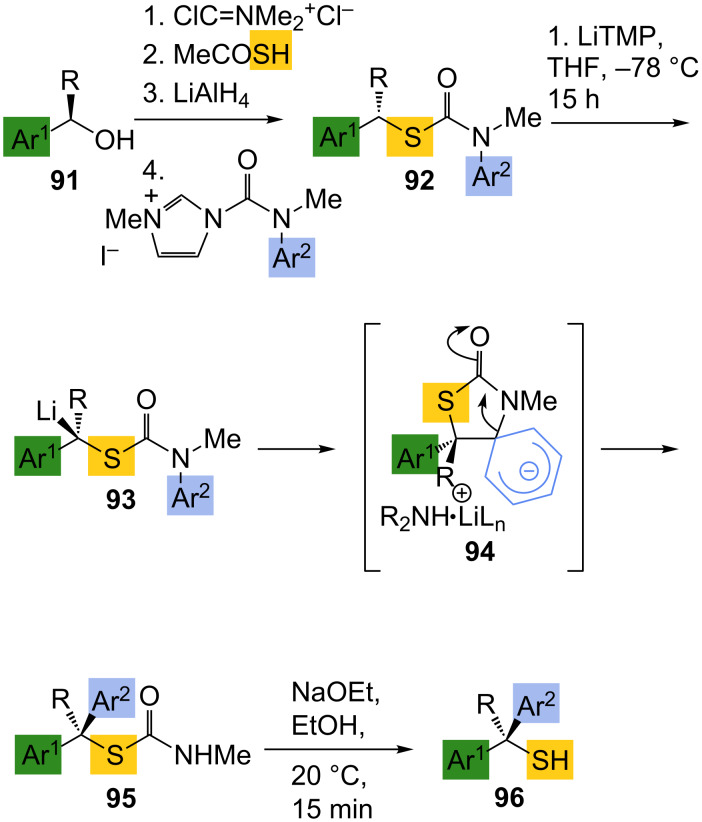
Asymmetric preparation of thiols **96** by aryl migration in lithiated thiocarbamates.

Carbon–carbon bond formation in configurationally stable α-thioorganolithiums allows access to non-racemic tertiary thiols of varying structure and complexity. Hoppe’s extensive studies of lithiated thiocarbamates have continued to display the synthetic utility of these species in the formation of secondary thiol derivatives. These were prepared by diastereoselective electrophilic additions in proline-derived systems [71–72] and asymmetric alkylation of thiocarbamates in the presence of a chiral ligand [73–74]. Stereospecific functionalisation of configurationally stable lithiated thiocarbamates with electrophiles is general for a range of structures, but until very recently it has not been applicable to *arylation* reactions. However, we have discovered [75] a variant of a rearrangement reaction of lithiated ureas that we first reported in 2007 [76] in which a lithiated thiocarbamate undergoes an intramolecular aryl transfer. Treatment of an *N*-aryl *S*-benzyl thiocarbamate **92** leads to formation of a lithio derivative **93** comparable with those reported by Hoppe (Scheme 32). As in the case of lithiated *N*-aryl ureas [76–80] and *N*-aryl carbamates [81–82], the *N*-aryl ring is susceptible to attack by the anionic centre, and migration of the ring to the position α to sulfur occurs, presumably via an intermediate related to **94**. After work up the product is a tertiary thiocarbamate **95**, and simple mildly basic hydrolysis leads to the tertiary thiol **96**. The aryl migration is applicable to a range of products **96** (Table 1) and amounts to an intramolecular arylation, allowing the formation of otherwise inaccessible doubly benzylic tertiary thiols in enantiomerically enriched form from benzylic alcohols **91**. Similar to the rearrangement of lithiated ureas [76] (but interestingly in contrast to the rearrangement of lithiated *carbamates* [81–82]), aryl migration proceeds with retention of stereochemistry. Current work is continuing with the aim of expanding the utility of this new mode of reactivity displayed by thiocarbamates.

**Table 1:** Scope of the thiocarbamate rearrangement [75].

SM, er	Ar^1^	Ar^2^	R	**95** yield (%)	**95** er

(*S*)-**92a**, 98:2	Ph	4-MeC_6_H_4_	Me	83	96:4
(*S*)-**92b**, 97:3	Ph	2-OMeC_6_H_4_	Me	89	91:9
(*S*)-**92c**, 98:2	Ph	4-ClC_6_H_4_	Me	94	96:4
(*S*)-**92d**, 98:2	Ph	3-ClC_6_H_4_	Me	69	96:4
(*S*)-**92e**, 98:2	Ph	4-CNC_6_H_4_	Me	78	97:3
(*S*)-**92f**, 99:1	Ph	1-naphthyl	Me	98	96:4
(*S*)-**92g**, 98:2	Ph	3-FC_6_H_4_	Me	0	–
(*R*)-**92h**, 99:1	Ph	4-MeC_6_H_4_	*n*-Pr	74	98:2
(*R*)-**92i**, 96:4	3-CF_3_	Ph	Me	85	67:33
(±)-**92j**, 50:50	4-OMe	4-MeC_6_H_4_	Me	86	50:50
(*R*)-**92k**, 79:21	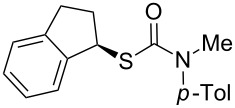			87	74:26

## Conclusion

Of the methods available for the synthesis of tertiary thiols, the majority (for example, those involving epoxide opening or electrophilic attack on thio-substituted enolates) lead to thiols carrying further functionality in some form. Tertiary thiols without further functionality are still challenging, with organolithium-based alkylations and arylation (by rearrangement) of thiocarbamates offering the best prospects. Further developments in this area – particularly with regard to carbolithiation reactions [79] – are to be expected.
